# Fractal Analysis of DNA Sequences Using Frequency Chaos Game Representation and Small-Angle Scattering

**DOI:** 10.3390/ijms23031847

**Published:** 2022-02-06

**Authors:** Eugen Mircea Anitas

**Affiliations:** 1 Joint Institute for Nuclear Research, 141980 Dubna, Russia; anitas@theor.jinr.ru; 2 Horia Hulubei, National Institute of Physics and Nuclear Engineering, 077125 Bucharest, Romania

**Keywords:** DNA sequences, time series, chaos game representation, multifractals, small-angle scattering

## Abstract

The fractal characteristics of DNA sequences are studied using the frequency chaos game representation (FCGR) and small-angle scattering (SAS) technique. The FCGR allows representation of the frequencies of occurrence of *k*-mers (oligonucleotides of length *k*) in the form of images. The numerically encoded data are then used in a SAS analysis to enhance hidden features in DNA sequences. It is shown that the simulated SAS intensity allows us to obtain the fractal dimensions and scaling factors at various scales. These structural parameters can be used to distinguish unambiguously between the scaling properties of complex hierarchical DNA sequences. The validity of this approach is illustrated on several sequences from: *Escherichia coli, Mouse* mitochondrion, *Homo sapiens* mitochondrion and *Human cosmid*.

## 1. Introduction

Recent advances in molecular biology technologies, such as nanopore sequencing, have enabled researchers to sequence and assemble very large genomes with high accuracy and throughput [1]. The landmark achievement is the release of the complete sequence of a human genome [2,3], with a total sequence length of around 3.1 gigabase pairs (Gb). For animals, it has been found that the genome length can extend well beyond this value, with 32 Gb for the axolotl genome [4] and 43 Gb for the Australian lungfish’s genome [5].

The nucleotide’s sequence provided by the full genomes is essential for understanding the biology and evolution of organisms. For humans, the full genome may provide important information about the chromosome function, genomic variation or human diseases [2]. Therefore, extracting meaningful biological and medical insights from these sequences requires approaches that take into account both local and global compositional heterogeneity. Locally, *short-term correlations* are always present in most coding sequences [6], and small changes in their structure may have significant effects on the evolutionary properties of the organism, its behavior, physiology, anatomy, etc. [7]. Globally, nucleotide sequences are also *long-range correlated*, i.e., nucleotides situated hundreds/thousands of base pairs apart from each other are known to be correlated [8,9].

Theoretically, both short- and long-range correlations can be appropriately captured through a power-law distribution [10,11,12]. Therefore, fractal concepts and scale invariance become important tools for the analysis of the structural complexity of nucleotide sequences. To this aim, two main approaches are used to determine the fractal dimension or the scaling exponents on finite scaling ranges. The first one involves the representation of a nucleotide sequence as a numerical series, followed by subsequent analysis. The most common methods are wavelet-based analysis [13], random walk and gap plots [14], DNA walks [10] or multifractal detrended fluctuation analysis [15]. The second one involves the representation of a nucleotide sequence as an abstract string of symbols [16,17]. These are known under the collective name of chaos game representation (CGR), and allow a visual representation of local and global patterns in a sequence. Thus, due to the complementarity of these two approaches, quite often, a DNA sequence analysis is performed using both numerical and abstract representation [7,18,19].

Although a visual characterization of patterns allows the recognition of features and judging of their similarities, an objective, mathematical measure is needed [16]. To this aim, multifractal analysis of CGR images and analysis of corresponding small-angle scattering (SAS) patterns have been relatively recently proposed to formalize similarities between patterns [7,19,20]. A multifractal analysis allows us to characterize the spatial inhomogeneity, and can reveal the existence of evolutionary information, which can be further applied to phylogenetic studies [7,20]. However, for some types of point distributions arising from CGR images, the multifractal spectra may be hardly distinguishable, and it has been shown that SAS formalism can address this issue, by providing additional structural information (i.e., scaling factor and fractal iteration number) [19].

Here, the approach introduced in ref. [19] is extended, and an SAS is simulated on a frequency chaos game representation (FCGR) of nucleotide sequences in order to extract the fractal dimensions and scaling factors *at various scales*. A detailed analysis is presented for *Escherichia (E.) coli, Mouse (M.)* mitochondrion, *Homo (H.) sapiens* mitochondrion and *Human (H.) cosmid* sequences, and for two artificial sequences, serving as a benchmark. In the latter case, one sequence is represented by a repetition of a group of four nucleotides, and the second one by a random distribution of nucleotides. To illustrate the validity of the new FCGR-SAS approach, for all six sequences, results from fluctuation and multifractal analyses are included.

## 2. Theoretical Background

### 2.1. CGR Representations of Sequences

Chaos game representation (CGR) is an iterative mapping technique that allows a graphical representation of DNA sequences in the form of an image. The standard algorithm for this conversion was initially presented in ref. [16], and it involves the following steps:

1. The four letters “A”, “T”/“U”, “G” and “C” composing the sequence are placed at the vertices of a square centered in the origin.
2. The first nucleotide in the sequence is placed at the midpoint between the center of the square and the vertex denoted by the same letter as the first nucleotide.
3. The position of the second nucleotide is obtained by placing it at the midpoint between the position of the first nucleotide and the vertex square denoted by the same letter as the second nucleotide.
4. The positions of each subsequent nucleotide are obtained as the midpoint between the position of the previous nucleotide and the vertex square corresponding to the current nucleotide.

In this representation, each point of CGR corresponds to exactly one subsequence (starting from the first base), and the whole original nucleotide subsequence up to the current nucleotide can be reconstructed just by knowing the corresponding point in CGR representation.

**Figure 1:**
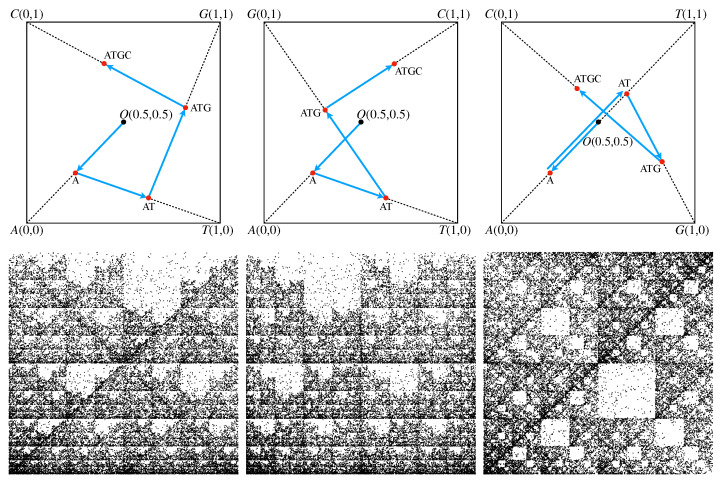
(**Upper row**) CGR for the short sequence “ATGC” in different configurations of the bases (see main text for details). (**Left**) RY configuration. (**Middle**) MK configuration. (**Right**) WS configuration. The arrow from nucleotide “A” to the sequence “AT” has been slightly shifted vertically for clarity. (**Lower row**) CGR for the *H. cosmid* g1346a094, GenBank ID: AC000362.1 (40,977 bp) in different configurations of the bases. (**Left**) RY configuration. (**Middle**) MK configuration. (**Right**) WS configuration.

An illustration of this algorithm for the simple sequence “ATGC” is provided in Figure 1 (upper row) for three possible configurations of a square’s vertices. Each configuration is based on the chemical structure and the strength of the hydrogen bond. In the first configuration shown in Figure 1 (upper row—Left), the elements of the minor diagonal are purine R = {A, G}, while on the main diagonal are fixed pyrimidine elements Y = {C, T}, hence the name RY configuration. In the second configuration, shown in Figure 1 (upper row—middle), one considers that the elements of the amino group M = {A, C} and of the keto group K = {G, T} are placed on the minor, and respectively on the main diagonals, hence the name MK configuration. In the third configuration, shown in Figure 1 (upper row—right), one considers that the elements of weak hydrogen bonds W = {A, T} and of strong hydrogen bonds S = {C, G} are placed on the minor, and respectively on the main diagonals, hence the name WS configuration.

Thus, the CGR algorithm is applied as follows: the first nucleotide “A” is placed at the midpoint between the square origin and the vertex “A”. The second nucleotide “T” is placed at the midpoint between the position of “A” and the vertex “T”, thus giving the position of the subsequence “AT”. The third nucleotide “G” is placed at the midpoint between the position of “AT” and the the vertex “G”, thus giving the position of subsequence “ATG”. Finally, the fourth nucleotide “C” is placed at the midpoint between the position of subsequence “ATG” and square vertex “C”.

Since every configuration has a different ordering of vertices, the resulting CGR representation is different. However, for long sequences, the CGR representation exhibits the property of self-similarity, i.e., a given pattern is repeated at different scales, for all three types of configurations. Figure 1 (lower row) shows this property for the *H. cosmid* g1346a094, GenBank ID: AC000362.1 (40,977 bp). This suggests that fractal theory is the appropriate framework to analye such structures, since it reveals the scaling properties.

In the rest of the paper, we present results for the RY configuration, as initially introduced by Jeffrey in ref. [16]. By considering the one-to-one correspondence between the CGR points and subsequences (see discussion above) and the fractal nature of the sequences, the presented analysis can be applied similarly to MK and WS configurations.

### 2.2. FCGR Representation of Sequences

An important application of CGR consists in evaluating the abundance of *k*-mers in a nucleotide sequence. This is performed graphically by representing the set of frequencies of *k*-mers within a given sequence in the form of an image, in which each pixel corresponds to a particular *k*-mer [16]. In the literature, this is known as FCGR; it allows nucleotide sequences amenable to an entire new set of statistical analysis tools, and it enables machine learning approaches to be applied [17].

**Figure 2:**
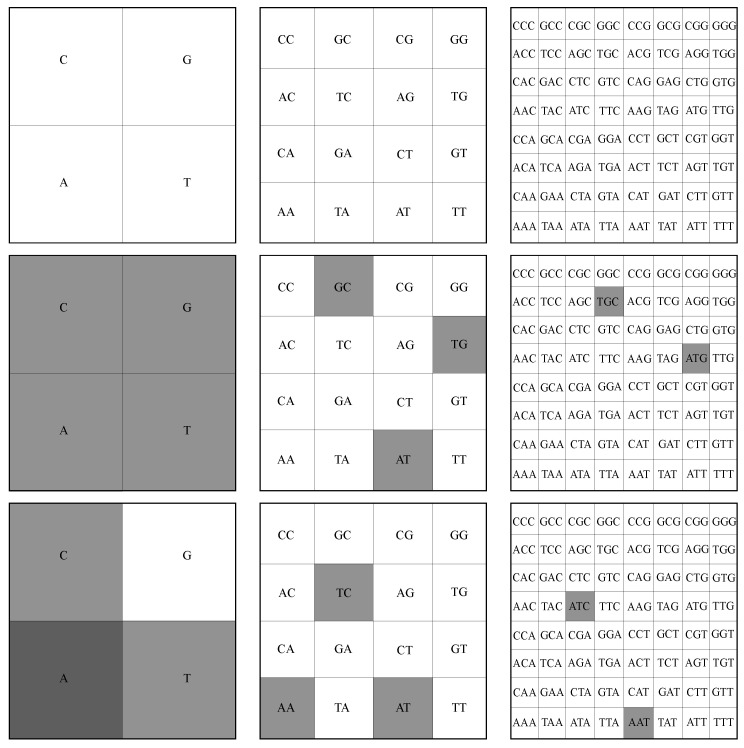
Quadrants in FCGR at different pixelation levels *k*, in which each quadrant corresponds uniquely to a specific string of length *k*. (**Left column**) k=1. (**Middle column**) k=2. (**Right column**) k=3 (see text for details). (**Upper row**) Construction of quadrants, sub-quadrants and sub-sub-quadrants. (**Middle row**) FCGR representation of “ATGC” sequence. (**Bottom row**) FCGR representation of “AATC” sequence.

For illustration, let us consider that the original square in Figure 1 (upper row—left) is divided into four quadrants (i.e., pixelation level of 1). This is shown in Figure 2 (upper row—left), where A is called the lower-left quadrant, T is the lower-right quadrant, G the upper-right quadrant and C the upper-left quadrant. As such, each *k*-mer of length 1 in a given sequence shall belong to one of these four quadrants. For the “ATGC” sequence used above, we obtain 1 point in each quadrant since we have exactly four different letters. This is also clear from Figure 1 (upper row—left). FCGR counts the occurrence of monomers in each quadrant and assigns a relative grayscale value. Generally, the higher the number of occurrences (i.e., the frequency), the darker the quadrant and vice versa. Therefore, for the “ATGC” string, each corresponding quadrant is represented by the same gray level (Figure 2 (middle row—left)). For a different sequence, such as “AATC”, we have 2 points in the A quadrant, 1 point in the T quadrant, 1 point in the C quadrant and no points in the G quadrant. Thus, the gray level of the A quadrant is twice as high as the one of the T and C quadrants, while the G quadrant is white, as shown in Figure 2 (lower row—left).

The FCGR for dimers is obtained by further dividing each quadrant into four similar sub-quadrants (i.e., pixelation level of 2), as shown in (Figure 2 (upper row—middle)). For a given quadrant, each of the 4 sub-quadrants contains sequences ending with a given dimer, and they differ only in the first letter. As in the case of monomers (see discussion above), the frequencies within each sub-quadrant are computed and are displayed by the intensity of the gray levels of each sub-quadrant. For the “ATGC” and “AATC” sequences, the FCGR tables are shown in Figure 2 (middle row—middle), and respectively in Figure 2 (lower row—middle). In both cases, the frequencies of occurrences are the same for each dimer and thus all intensity levels are the same.

Generally, to obtain points representing nucleotides of length *k*, we continue the above procedure up to a pixelation level of *k*. Figure 2 also shows the construction rule of sub-sub-quadrants for trimers (upper row—right), and the corresponding tables for “ATCG” (middle row–right) and “AATC” sequences (lower row—right) at a pixelation level of 3. Note that the FCRG tables contain all possible combinations of nucleotide letters of a given length, and generally only a fraction of them are “occupied”. In particular, the higher the pixelation level, the lower the “occupancy” level. Moreover, from construction, it results that if the pixelation level is higher than the length of the nucleotide, the FCGR table shall be completely empty.

### 2.3. Time Series and Fractal Theory

A fractal analysis is performed on the time series model, similar to the one described in ref. [21]. This involves defining a map *g* over the alphabet {A, C, G, T} such that one can distinguish A and G from purine, and C and T from pyrimidine. Formally, we can write this as: g(A)→2, g(T)→−1, g(G)→1, g(C)→−2. This gives a sequence $\left\{x_{k}:k=1,\cdots ,N\right\}$, with $x_{k}\in \left\{\pm 1,\pm 2\right\}$, and which is interpreted as a time series x(t). Here, *N* is the length of the sequence.

Long memory processes can be revealed by starting from a detrended fluctuation analysis (DFA) [22]. To this aim, one can use the scaling properties of the fluctuation function F(n), obtained from the cumulative sum of the process x(t). Here, *n* is the length of the $N_{n}$ non-overlapping time intervals of $y\left(t\right)\equiv \sum_{m=1}^{t}\left[x\left(m\right)-\langle x\rangle \right]$, where 〈x〉 is the mean of x(t). To account for possible data loss arising from incomplete coverage of y(t), the same procedure is repeated starting from its end, thus giving rise to a total of $2N_{n}$ intervals. Then, for each interval *j*, $j=1,\cdots ,2N_{n}$, all data are fitted with a polynomial $y_{j}$, and the corresponding variance is computed as [15]:

(1) $$F{\left(n,j\right)}^{2}=\{\begin{array}{c} \frac{1}{n}\sum_{i=1}^{n}\;{\left[y\left(\left(j-1\right)n+i\right)-y_{j}\left(i\right)\right]}^{2},\;\;\;j=1,\cdots ,N_{n}\\ \frac{1}{n}\sum_{i=1}^{n}\;{\left[y\left(N-\left(j-N_{n}\right)n+i\right)-y_{j}\left(i\right)\right]}^{2},\;\;\;j=N_{n}+1,\cdots ,2N_{n}. \end{array}$$

The multiple scaling properties of $F_{n}$ are taken into account by performing a multifractal detrended fluctuation analysis (MFDFA), i.e., the fluctuation function is computed at different orders of q∈R [15], i.e.,

(2) $$F_{q}\left(n\right)={\left[\frac{1}{2N_{n}}\sum_{j=1}^{2N_{n}}{\left[F^{2}\left(n,j\right)\right]}^{q/2}\right]}^{1/q}\propto n^{h(q)},$$

where h(q) is called the generalized Hurst exponent. Thus, for q>0 will dominate intervals *j* with large variance $F^{2}\left(n,j\right)$, while for q<0 will dominate intervals with small variance. This scaling behavior of time intervals with both large and small fluctuations can be determined from the behavior of h(q).

To better describe the different scalings of the time series, one calculates the multifractal spectrum according to [15]:

(3) f(α)=q[α−h(q)]+1,

where $\alpha =h\left(q\right)+q\frac{dh(q)}{dq}$,

### 2.4. Small-Angle Scattering Technique

The SAS intensity is calculated by extracting first the position vectors $r_{i}$ and the corresponding gray levels $b_{i}$, with $i=1,\cdots ,2^{2k}$ of each pixel from the CGR image. Here, *k* is the pixelation level, and the origin of the square is chosen at the “A” corner. In an SAS experiment, $b_{i}$ corresponds to the scattering length attached to each atom at position $r_{i}$.

Thus, the normalized amplitude of elastic scattering by an ensemble containing $2^{2k}$ atoms becomes:

(4) $$A\left(q\right)={\left(\sum_{i=1}^{2^{2k}}b_{i}\right)}^{-1}\sum_{i=1}^{2^{2k}}b_{i}e^{q\cdot r_{i}}.$$

Here, $q=4\pi \lambda^{-1}\sin\theta$ is the modulus of the scattering vector q, θ is the incident angle, and λ is the wavelength of the incident radiation. Then, the scattering intensity is obtained as the product of the amplitude with its complex conjugate, averaged over all orientations of q, i.e.,

(5) $$I\left(q\right)\equiv \langle A\left(q\right)A^{*}\left(q\right)\rangle$$

Here, the brackets $\langle \cdots \rangle$ stand for the ensemble averaging, and for an arbitrary function *f*, it is calculated according to $\langle f(q_{x},q_{y})\rangle ={\left(2\pi \right)}^{-1}\int_{0}^{2\pi }f\left(q,\phi \right)d\phi$, where $q_{x}=q\cos\phi$ and $q_{y}=q\sin\phi$ are the components of q in a polar coordinate system. From the definition (Equation (5)), the scattering intensity obeys the condition I(0)=1.

Generally, for exact self-similar fractals such as those arising from CGR, the SAS intensity is characterized by a superposition of maxima and minima superimposed on a power-law decay with the exponent related to the fractal dimension of the sequence [19]. In order to smooth such curves, we consider an ensemble of “ATGC” squares of different sizes *l*, and define a distribution function $D_{N}\left(l\right)$ of sizes such that $D_{N}\left(d\right)dl$ is the probability of finding a square whose size is in the range (l,l+dl). Here, a log-normal distribution is considered, such as:

(6) $$D_{N}\left(l\right)=\frac{1}{\sigma l{\left(2\pi \right)}^{1/2}}\exp\left(-\frac{{\left[\log\left(l/\mu_{0}\right)+\sigma^{2}/2\right]}^{2}}{2\sigma^{2}}\right),$$

where $\sigma ={\left[\log\left(1+\sigma_{r}^{2}\right)\right]}^{1/2}$. The quantities $\mu_{0}$ and $\sigma_{r}$ are the mean length and relative variance, i.e., $\mu_{0}\equiv {\langle l\rangle }_{D}$ and $\sigma_{r}\equiv {\left({\langle l^{2}\rangle }_{D}-\mu_{0}^{2}\right)}^{1/2}/\mu_{0}$ and $\langle \cdots \rangle \equiv \int_{0}^{\infty }\cdots D_{N}\left(d\right)dl.$

Since the positions of squares are uncorrelated, the SAS intensity in the presence of polydispersity is calculated as the average of Equation (5) over the distribution ($D_{N}$), i.e.,

(7) $$I_{\mathrm{poly}}\left(q\right)=\int_{0}^{\infty }I\left(q\right)D_{N}\left(l\right)dl.$$

An important feature of SAS from fractals is its ability to differentiate between “mass” and “surface” fractals [23]. This is reflected in the value of the scattering exponent τ of the SAS intensity (Equation (7)), i.e.,

(8) $$I\left(q\right)\propto q^{-\tau },$$

where $\tau =D_{m}$ for mass fractals, and $\tau =2d-D_{s}$ for surface fractals. Here, *d* is the Euclidean dimension of the space in which the fractal is embedded, $0<D_{m}<d$ is the mass fractal dimension, and $d-1<D_{s}<d$ is the surface fractal dimension. For two-dimensional fractals, such as those resulting from FCGR, we have d=2; thus, if, in an SAS curve, the measured exponent is τ<2, the structure is a mass fractal, and if 2<τ<3, the structure is a surface fractal.

## 3. Results and Discussion

### 3.1. Time Series Analysis

**Figure 3:**
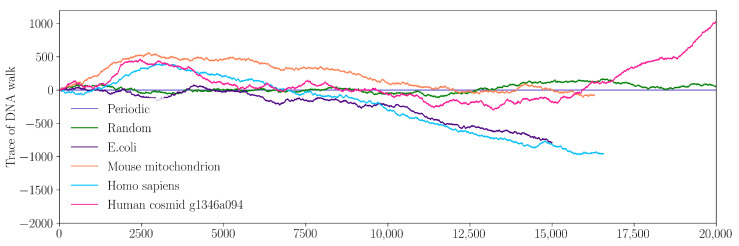
Time series representation of various sequences, as described in Section 2.3. (Light violet) Test sequence “ATGC” repeated 5 $\times \;10^{3}$ times. (Green) Test sequence consisting of $10^{6}$ nucleotides randomly distributed, and chosen in such a way that their frequencies of occurrence are approximately equal. (Dark violet) *E. coli*, O145:H28 162405, sequence 161, GenBank ID: BJSS01000161 (15,000 bp). (Orange) *M.* mitochondrion, complete genome, GenBank ID: 342520 (16,295 bp). (Light blue) *H. sapiens* mitochondrion, complete genome NCBI Reference Sequence: NC_012920.1 (16,569 bp). (Pink) *H. cosmid* g1346a094, GenBank ID: AC000362.1 (40,977 bp).

A time series representation for four DNA sequences is shown in Figure 3: *E. coli* (dark violet), *M.* mitochondrion (orange), *H. sapiens* (light blue) and *H. cosmid* (pink). For comparison, time series for two artificial sequences are also shown: a periodic sequence in which the string “ATGC” is repeated $5\times 10^{3}$ times (light violet), and a sequence consisting of all letters “A”, “T”, “G” and “C” randomly distributed (green). As expected, the time series representation of the periodic string may be assimilated to a straight line, while, for the random string, we have relatively small variations around this straight line. This allows us to visually assess that the *E. coli* sequence is closest to the random one, while the *H. cosmid* one shows the greatest variability.

### 3.2. Multifractal Detrended Fluctuation Analysis

**Figure 4:**
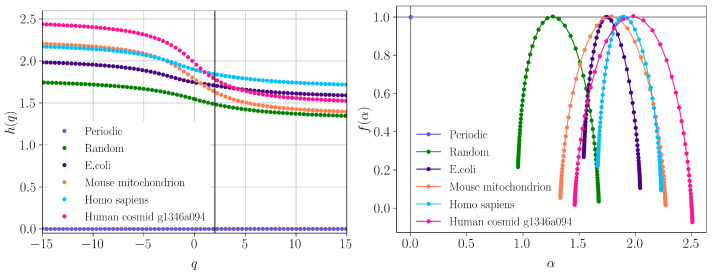
MFDFA analysis of DNA sequences. (**Left**) Generalized Hurst exponents h(q). The vertical line at q=2 helps to visualize the values h(2). They are in very good agreement with those obtained from the variation logF(n) vs. logn as shown in Figure 4. (**Right**) f(α) spectra, where α are scaling indices.

The generalized Hurst exponents h(q) for all the above six sequences are shown in Figure 4 (left). For the periodic sequence, h(q)=0 for all values of *q* (light violet), and it reveals a non-fractal behavior, as expected. The h(q) spectra for the complete random sequence (green), *E. coli* (dark violet) and *H. sapiens* mitochondrion (light blue) show relatively small variation with *q*. This indicates a simple fractal structure with long-range power-law correlations between nucleotides, which can be described by the presence of a small number of scaling factors. The h(q) spectra for *M.* mitochondrion (orange) and *H. cosmid* (pink) show more pronounced variation with *q*. This indicates a more heterogeneous sequence characterized by a well-defined multifractal structure with long-range power-law correlation between nucleotides, and with a relatively higher number of scaling factors.

The degree of correlations can be quantified from the value of the Hurst exponent *H* by using H=h(2) for stationary series and H=h(2)−1 for non-stationary series [24]. For each sequence, *H* corresponds to the intersection of the vertical (black) line at q=2 with the corresponding h(q) curves in Figure 4 (left). For the periodic sequence “ATGC” used here, H=0 and it indicates a time series with long-term switching between high and low values in adjacent pairs. According to the map *g* defined in Section 2.3, the values of the corresponding periodic series are “2, 1, 2, 0, 2, 1, 2, 0, ⋯”. For the random sequence, one obtains H≲0.5 and reveals a weakly correlated nucleotide sequence. For the other sequences, one obtains 0.7≲H≲0.85, which indicates sequences with long-term autocorrelation, with higher values showing less pronounced roughness and volatility and a smoother trend [25].

The corresponding f(α) spectra obtained from Equation (3) are shown in Figure 4 (right). Except for the periodic sequence, for which the spectrum degenerates to a single point (with coordinates (0,1)), all the other curves show concave behavior with maxima at scaling indices α=h(2). The width of f(α) is a measure of the degree of multifractality: the greater the width, the more heterogeneous the fractal, and vice versa. As such, *H. cosmid* appears to be the most heterogeneous sequence. The asymmetry of each f(α), i.e., the presence of a shorter left branch as compared to the right one, results from the higher contribution of moments with q<0, i.e., small fluctuations within nucleotide sequences are slightly more pronounced as compared to large ones.

### 3.3. FCGR Analysis

**Figure 5:**
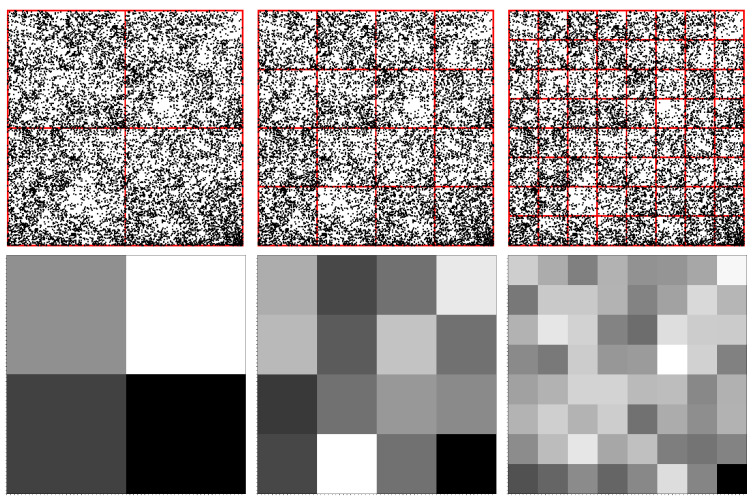
Two representations of *E. coli* in the “ATGC” system. (**Upper row**) CGR, where the positions of the nucleotides are in orange. On top of each image is superimposed a grid of different sizes: 2 × 2 (**left**), 4 × 4 (**middle**) and 8 × 8 (**right**) cells. (**Lower row**) FCGR for monomers (**left**), 2-mers (**middle**) and 3-mers (**right**).

The construction of FCGR images, as described in Section 2.2, is illustrated for a long nucleotide sequence (*E. coli*) in Figure 5, for k=1,2,3. For k=1, the CGR image is divided into four quadrants in Figure 5 (upper row—left). For better visualization, the edges of the four quadrants are marked in red. The number of points in each quadrant is recorded: the quadrant with the highest number is assigned the black color, and the other quadrants are assigned gray levels proportional to the respective number of points (lower row—left). For k=2 and k=3, the same procedure is repeated, and the configurations shown in the middle column and respectively in the right column are obtained. Thus, the abundance of nucleotide sequences within the image is reflected through the various levels of gray: the higher the *k*, the larger the variability, and vice versa.

**Figure 6:**
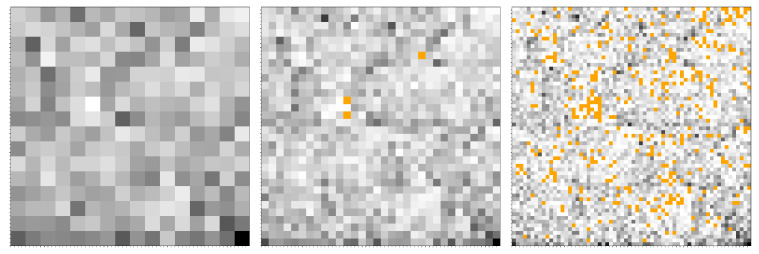
FCGR of *E. coli* at various iterations *k*. (**Left**) *k* = 4. (**Middle**) *k* = 5. (**Right**) *k* = 6. Orange points indicate missing nucleotide positions.

However, when *k* reaches a threshold $k_{\min}$ dependent on the type of the sequence, not all possible combinations of nucleotides of length $k_{\min}$ may be present. As such, the corresponding sub-quadrants at pixelation levels $k>k_{\min}$ are left empty, and they are not counted when the SAS intensity is calculated, i.e., one considers $b_{i}=0$ in Equation (5), where *i* spans the set of empty sub-quadrants. According to the FCGR representation described above, they correspond to a white color. However, since the abundance of many nucleotides is close to zero, the corresponding gray levels are small, and therefore hardly distinguishable from empty sub-quadrants. Therefore, here, the positions of missing nucleotides are represented in orange. For illustration, Figure 6 shows the FCGR of *E. coli* at k=4 (left), k=5 (middle) and k=6 (right). For this sequence, all possible combinations of nucleotides are present up to k=4. At $k=5\;(\equiv k_{\min})$, there are three missing nucleotides, and at k=6, their number increases significantly, as expected. Obviously, in the limit k=N (where N is the sequence length), one obtains a single point, while all others are empty.

**Figure 7:**
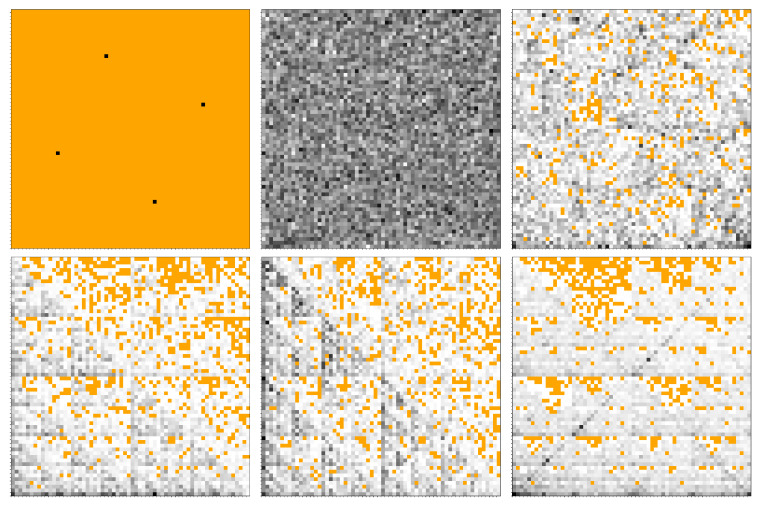
FCGR of DNA sequences at k=6. (**Upper left**) Periodic DNA. (**Upper middle**) Random DNA. (**Upper right**) *E. coli*. (**Lower left**) *M.* mitochondrion. (**Lower middle**) *H. sapiens*. (**Lower right**) *H. cosmid* g1346a094.

The FCGR for the same six sequences used in Figure 3 and Figure 4 is shown in Figure 7 at pixelation level k=6. For the periodic sequence “ATGC”, the FCGR reduces to four regions of equal probabilities (black), and all others are empty (orange; upper left). For the random sequence, the FCGR shows a random-like distribution for all gray levels across the whole image, indicating no correlations between the positions of nucleotides, as expected (upper middle). At this pixelation level, all possible combinations of nucleotide sequences are present, since there are no orange sub-quadrants. This is due to the much larger number of nucleotides ($10^{6}$) used, as compared to the other sequences (see also the caption of Figure 3). For *E. coli*, the distribution of gray levels is quasi-random, as indicated by the presence of regions with slightly higher gray levels (mostly near the “T” vertex) compared to other regions. However, for the *M.* mitochondrion (lower left), *H. sapiens* mitochondrion (lower middle) and *H. cosmid* (lower right), one can observe repeating patterns at various scales, indicating the presence of long-range correlations. Note that, for a given scale, the distribution of gray levels is not uniform, revealing the heterogeneity of the nucleotides’ distribution, as discussed also in Section 3.2. As will be shown below, a quantification of this heterogeneity in terms of fractal dimensions and scaling factors at various scales can be provided by an SAS analysis. Note that regions of missing subsequences in the *M.* mitochondrion, *H. sapiens* mitochondrion and *H. cosmid* also resemble partially ordered structures, or even fractal-like organization for *H. cosmid*.

### 3.4. Small-Angle Scattering Analysis

**Figure 8:**
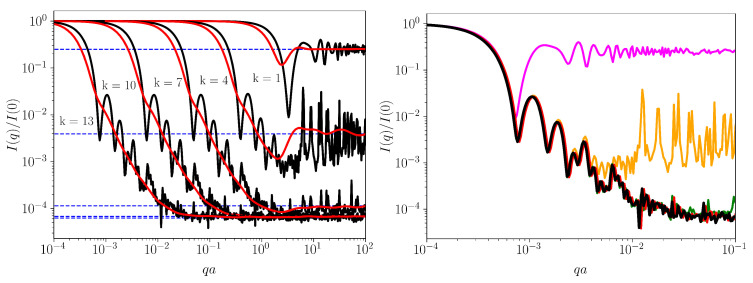
SAS from *E. coli* at pixelation levels k=1,4,7 and 13. (**Left** (Black) Monodisperse intensity (Equation (5). (Red) Polydisperse (smoothed) intensity (Equation (7)). (Dashed blue) asymptotic values at high *q*. Curves for k<13 are shifted horizontally for better visualization). (**Right**) The same curves but not shifted. (Pink) k=1. (Orange) k=4. (Green) k=7. (Red) k=10. (Black) k=13.

Once the position vectors **r**${}_{i}$ and gray levels $b_{i}$ for non-empty sub-quadrants are extracted from FCGR, the monodisperse and polydisperse SAS intensities can be calculated according to Equation (5), and respectively with Equation (7). The results for *E. coli* are shown in Figure 8 (left) in black (moonodisperse SAS) and red (polydisperse SAS) at k=1,4,7,10 and 13. For better visualization, the curves corresponding to k<13 are shifted horizontally. For every *k*, the main feature of the monodisperse curves is the presence of three regions. For example, at k=10, we have $I\left(q\right)\propto q^{0}$ when $qa≲2\times 10^{-3}$ (called Guinier region), where *a* is a measure of the overall size of the “ATGC” square. The upper bound for qa provides information about the size of this square, so, for our purposes, it has no significance, since we can arbitrarily choose the position of square vertices, without affecting FCGR. When $2\times 10^{-3}≲qa≲2\times 10^{-1}$, we have a superposition of maxima and minima on a succession of power-law decays. Within each *q*-range spanned by a single power-law decay, the presence of these maxima and minima is a signature of exact self-similarity, i.e., regions inside FCGR contain parts that are *exact* replicas of the whole. Thus, their periodicity can be used to asses the scaling factors specific to the nucleotide sequence [26]. The corresponding polydisperse curve has a smooth behavior and allows us to assess the fractal dimensions [23]. The upper bound of this range is related to the smallest distance between non-empty sub-quadrants (i.e., individual pixels), for mass fractals, and to the size of the smallest sub-quadrant, for surface fractals. Finally, when $qa≳2\times 10^{-1}$, we have an asymptotic region, where $I\left(q\right)\propto 1.1\times 10^{-4}$ (blue-dashed line), which gives the total number of non-empty sub-quadrants. For arbitrary *k*, in the asymptotic region, I(q)≃1/N, as seen by the position of the blue-dashed lines in Figure 8 (left).

The unshifted monodisperse curves are shown in Figure 8 (right) in pink (k=1), orange (k=4), green (k=7), red (k=10) and black (k=13). This clearly reveals that by increasing *k*, the length of the *q*-range over which maxima and minima are superimposed also increases. The increase takes place such that a curve at a given pixelation level *k* completely reproduces the curves at smaller pixelation levels up to the beginning of their asymptotic region. For example, at k=7, the green curve reproduces the orange one (k=4) up to $qa≲6\times 10^{-3}$. For higher values, the green curves continues to decay with additional maxima and minima up until $qa≲4\times 10^{-2}$, when its asymptotic region begins. Generally, the differences between two curves at pixelation levels k+1 and *k* arise due to the fact that, in the former case, one considers also nucleotides of greater length. Depending on how these subsequences (of length k+1) are distributed, they may give rise to various types of decay of SAS intensity. In particular, if there is a power-law correlation between them, the scattering exponent τ (see Section 2.4) is related to the fractal dimension at the corresponding scale.

As discussed in Section 3.3, by increasing *k*, the number of empty sub-quadrants increases (see Figure 6 and Figure 7), and thus the rate of increase in non-empty sub-quadrants decreases. This leads to smaller and smaller differences between scattering curves with increasing *k*. For the sequence of *E. coli* used here, when k>10, the differences are hardly distinguishable, as indicated by the proximity of the two lowermost blue-dashed lines in Figure 8 (left) or by the almost complete superposition of the red and black curves in Figure 8 (right).

**Figure 9:**
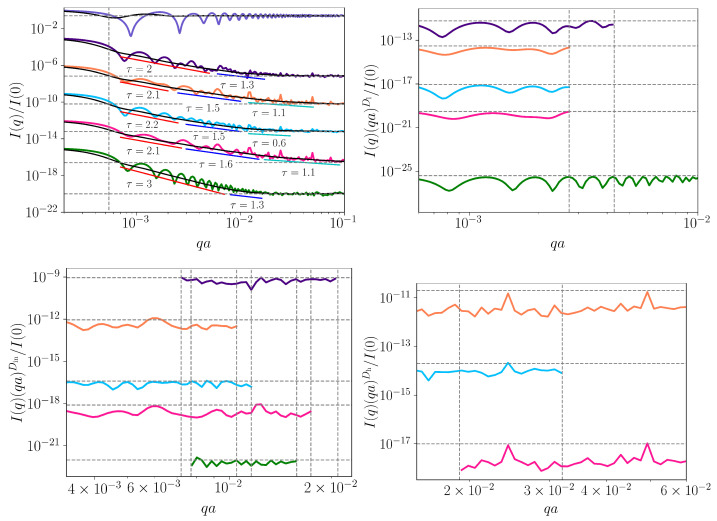
(**Upper left**) SAS intensities (Equation (5)) from periodic “ATGC” sequence (violet), random sequence (green), *E. coli* (dark violet), *M.* mitochondrion (orange), *H. sapiens* mitochondrion (light blue) and *H. cosmid* (pink) at k=13. Black lines: polydisperse SAS intensities (Equation (7)). Horizontal dashed lines: the asymptotic values. Vertical dashed lines: end of Guinier region and beginning of the power-law decay (see text for details). τ represents the scattering exponent. *a* is a measure of the “ATGC” square. All curves, except the periodic one, are shifted vertically down for better visualization. (**Upper right**, **lower left**, **lower right**) the functions $I\left(q\right){\left(qa\right)}^{\tau }$ vs. *q* on the first, second and third power-law decays with $\tau =D_{l}$, $\tau =D_{\mathrm{in}}$ and $\tau =D_{h}$, respectively, used to emphasize the periodicity of SAS intensities. Here, $D_{l}$ is a surface fractal dimension, while $D_{\mathrm{in}}$ and $D_{h}$ are mass fractal dimensions (see text for details). Vertical dashed lines represent the end of the fractal region for each nucleotide.

However, for sequences with lengths much greater than that of *E. coli*, we expect also that the intensities are hardly distinguishable at k≫10. Figure 9 (upper left) shows the monodisperse SAS intensities at k=13 for the six sequences analyzed in Figure 3, Figure 4 and Figure 7: the periodic “ATGC” sequence (violet), random sequence (green), *E. coli* (dark violet), *M.* mitochondrion (orange), *H. sapiens* mitochondrion (light blue) and *H. cosmid* (pink). Since the longest naturally occurring sequence used here (i.e., *H. cosmid*) is around 2.2 times longer than *E. coli*, this value of *k* will be sufficient to reveal the scattering properties at a few scales. The figure shows also the polydisperse intensities, all in black. Note that the intensities for all sequences, except the periodic one, are shifted vertically down for better visualization.

The results in Figure 9 show that although the periodic sequence “ATGC” is much longer, it has no structural correlations. This occurs since the FCGR (Figure 7 (upper left)) contains four sub-quadrants, and, as a consequence, the asymptotic region is at 1/4, immediately following the Guinier region. The remaining sequences are characterized by a superposition of maxima and minima after the Guinier region, superimposed on a succession of two or more power-law decays, with different scattering exponents. Within the *q*-range of each power-law decay, there are present at least two pronounced minima and maxima.

In the first set of power-law decays, τ=2 for *E. coli*, τ=2.1 for *M.* mitochondrion, τ=2.2 for *H. sapiens* mitochondrion, τ=2.1 for *H. cosmid* and τ=3 for the random sequence. In this last case, the power-law decay corresponds to a 2D regular structure. Indeed, the FCGR (Figure 7 (upper middle)) shows an almost uniformly filled square without any correlations in the sub-quadrant positions. Thus, the remaining four sequences show a surface fractal-like structure with dimensions $D_{s}=2$ (*E. coli*), $D_{s}=1.9$ (*M.* mitochondrion), $D_{s}=1.8$ (*H. sapiens* mitochondrion) and $D_{s}=1.9$ (*H. cosmid*; see discussion after Equation (8)). This may be interpreted in terms of a power-law distribution of sub-quadrant sizes forming mass fractals of various iterations [27].

The second set of power-law decays have exponents $\tau (\equiv D_{m})=1.3$ for E.coli, $\tau (\equiv D_{m})=1.5$ for M.mitochondrion, $\tau (\equiv D_{m})=1.5$ for H.sapiensmitochondrion and $\tau (\equiv D_{m})=1.6$ for H.cosmid. These values correspond to mass fractals that describe sub-quadrants of similar gray levels forming moderately branched structures at a smaller scale. Note that, for the random sequence “ATGC”, we also have a short region with exponent $\tau (\equiv D_{m})=1.3$, revealing some weakly branched structures formed by sub-quadrants. This could be the reason for the weak multifractality shown by the random sequence in the MFDFA analysis (Figure 4). Finally, the *M.* mitochondrion, *H. sapiens* mitochondrion and *H. cosmid* show also a third set of power-law scattering decays with $\tau (\equiv D_{m})=1.1,0.6$ and 1.1, respectively. These values reveal the presence of loosely branched groups of sub-quadrants at an even smaller-scale.

Figure 7 (lower row—middle) clearly shows an exact repetition of triangular shapes below the main diagonal, at various scales (i.e., regions with the same fractal dimensions). In turn, each triangle at a given scale consists of smaller triangles and so on. The exact self-similarity property is revealed by plotting $I\left(q\right){\left(qa\right)}^{\tau }$ vs. *q* in the fractal regions (see [26]). This is illustrated in Figure 9 (upper right, lower left, lower right) for each of the three sets of the power-law decays discussed above (Figure 9 (upper right)). Then, the period on the logarithmic scale is $\log_{10}\left(1/\beta_{s}\right)$, where $\beta_{}s$ is the corresponding scaling factor. The range over which the curves are displayed coincides with the corresponding fractal range. This is marked in Figure 9 (upper left) by the length of the orange lines (for the first set of power-law-decays), blue lines (for the second set) and cyan lines (for the third set). In Figure 9 (upper right, lower left and lower right), the end of the fractal range is marked by vertical black dotted lines.

For the first set of power-law decays (Figure 9 (upper right)), the periodicity for each sequence is relatively well pronounced, indicating regions of sub-quadrants separated by similar distances, and inside which there are subregions separated by similar distances but scaled down by a factor of $\beta_{s}$, as shown in Figure 7 (lower right, lower middle, lower right). Note that the maxima and minima for the random sequence are superimposed on a power-law decay with exponent τ=3, and thus do not describe any structural organization, as discussed above. For the second and third sets of power-law decays (Figure 9 (lower left) and (lower right)), one can observe maxima and minima but with smaller amplitudes and not very well-defined periodicity. This indicates a fractal structure in which the exact self-similarity is partially lost, and the structure has features characteristic of statistically self-similar fractals, i.e., regions inside FCGR contain parts that are *approximately* similar to the whole. This can be clearly seen for the *E. coli* sequence (violet curve), where the maxima and minima are significantly smeared.

## 4. Conclusions

Three leading approaches, MFDFA, FCGR and SAS, have been combined for revealing the fractal characteristics of DNA sequences at various scales. The strengths and weaknesses of these methods are illustrated for six sequences: two artificially created ones (periodic and random) serving as control sequences, and four naturally occurring ones but with various types of structural organization of the nucleotides—a quasi-random sequence (*E. coli*), moderate multifractal sequence (*H. sapiens*) and two sequences with relatively higher multifractality (*M.* mitochondrion and *H. cosmid*).

While MFDFA can reveal the presence of multifractality, FCGR provides a powerful visualization of the associated nucleotide sequences. Here, it has been shown that SAS can extend one step further the fractal characterization, by providing a way to extract additional key structural parameters. This is achieved by employing a new procedure, namely calculating the SAS intensity from the FCGR image and assimilating each pixel’s gray level with the corresponding scattering length at each pixel position.

As such, it has been shown that this approach allows us to:

- Differentiate between sequences with power-law correlations and without them. This can be derived from the value of the scattering exponent τ in the power-law decay of SAS intensity, i.e., τ<3 for power-law correlated sequences, and τ=3 for uncorrelated sequences with uniform distribution of nucleotides.
- Differentiate between simple power-law correlations (i.e., mass fractals) and a superposition of power-law correlations over different ranges (i.e., surface fractals), for fractal sequences. This can also be derived from the value of the exponent in the power-law decay of SAS intensity: τ<2 for mass fractals and 2<τ<3 for surface fractals. In the former case, the corresponding fractal dimension resulting from the FCGR is $D_{m}=\tau$, while, in the latter case, $D_{s}=4-\tau$.
- Reveal the presence of a *succession* of power-law correlations at different scales.
- Reveal the scaling factors at each scale (in addition to the scattering exponents τ), i.e., how groups of sequences of certain length combine to form repeating patterns, for exact self-similar fractal sequences.

This information may be useful in the construction of phylogenetic trees, classification of genomic data or in the identification of regions in chromosomes. In practice, the suggested approach can be used to compare genomic sequences when only parts of the genomes are available.

However, for very long sequences of the order of mega-base-pairs or more, one may have a succession of many power-law decays in the SAS intensity, which could be impractical for manual analysis. Then, an automated analysis procedure, involving eventually a software code with a friendly graphical user interface that allows the exporting of the structural parameters (e.g., fractal dimensions, number of power-law decays, scaling factor, etc.) is desirable to handle such large sequences. This could be even more useful in the context of biological big data, where also the number of available decoded sequences increases. Thus, machine learning algorithms could be applied to reveal previously unseen insights between local and global heterogeneities of sequences, as well as their biological functions.

## Data Availability

Not applicable.
